# Seasonal Coronaviruses and Other Neglected Respiratory Viruses: A Global Perspective and a Local Snapshot

**DOI:** 10.3389/fpubh.2021.691163

**Published:** 2021-07-05

**Authors:** Sunčanica Ljubin-Sternak, Tomislav Meštrović, Ivana Lukšić, Maja Mijač, Jasmina Vraneš

**Affiliations:** ^1^Clinical Microbiology Department, Andrija Štampar Teaching Institute of Public Health, Zagreb, Croatia; ^2^Medical Microbiology Department, University of Zagreb School of Medicine, Zagreb, Croatia; ^3^Clinical Microbiology and Parasitology Unit, Zora Profozić Polyclinic, Zagreb, Croatia; ^4^University Centre Varaždin, University North, Varaždin, Croatia

**Keywords:** respiratory tract infections, seasonal coronavirus, rhinovirus, adenovirus, bocavirus, Croatia

## Abstract

Respiratory viral infections are the leading cause of morbidity and mortality in the world; however, there are several groups of viruses that are insufficiently routinely sought for, and can thus be considered neglected from a diagnostic and clinical standpoint. Timely detection of seasonality of certain respiratory viruses (e.g., enveloped viruses such as seasonal coronaviruses) in the local context can aid substantially in targeted and cost-effective utilization of viral diagnostic approaches. For the other, non-enveloped and year-round viruses (i.e., rhinovirus, adenovirus, and bocavirus), a continuous virological diagnosis needs to be implemented in clinical laboratories to more effectively address the aetiology of respiratory infections, and assess the overall impact of these viruses on disease burden. While the coronavirus disease 2019 (COVID-19) pandemic is still actively unfolding, we aimed to emphasize the persistent role of seasonal coronaviruses, rhinoviruses, adenoviruses and bocaviruses in the aetiology of respiratory infections. Consequently, this paper concentrates on the burden and epidemiological trends of aforementioned viral groups on a global level, but also provides a snapshot of their prevalence patterns in Croatia in order to underscore the potential implications of viral seasonality. An overall global prevalence in respiratory tract infections was found to be between 0.5 and 18.4% for seasonal coronaviruses, between 13 and 59% for rhinoviruses, between 1 and 36% for human adenoviruses, and between 1 and 56.8% for human bocaviruses. A Croatian dataset on patients with respiratory tract infection and younger than 18 years of age has revealed a fairly high prevalence of rhinoviruses (33.4%), with much lower prevalence of adenoviruses (15.6%), seasonal coronaviruses (7.1%), and bocaviruses (5.3%). These insights represent a relevant discussion point in the context of the COVID-19 pandemic where the testing of non-SARS-CoV-2 viruses has been limited in many settings, making the monitoring of disease burden associated with other respiratory viruses rather difficult.

## Introduction

Respiratory tract infections (RTIs) represent a major public health matter in developed and developing countries alike, being responsible for about 19% of all deaths among children younger than 5 years of age, as well as 8.2% of disability and premature mortality (1–4). According to the World Health Organization (WHO), RTIs are actually placed first when we measure burden of disease by using disability-adjusted life-years (DALYs). Furthermore, lower RTIs are the third leading cause of death in the world overall (4). They are predominantly caused by viruses, and although our focus is primarily on human respiratory syncytial virus (RSV) in children and influenza viruses in adults, there is an underappreciated burden of RTIs caused by several viral groups, further compounded by the ongoing pandemic of coronavirus disease 2019 (COVID-19). There are various reasons why many respiratory viruses are not routinely sought for, and can thus be considered neglected from diagnostic and clinical standpoint. Moreover, due to the current lack of vaccines for many neglected respiratory viruses, which may change in not so distant future, a better grasp of their prevalence (especially in children), distribution and seasonality is indispensable for effective prevention, control, and treatment endeavours (5). This review aims to provide an outline of four viral groups (seasonal coronaviruses, rhinoviruses, adenoviruses, and bocaviruses), concentrating on epidemiological trends worldwide and estimating/summarizing the overall infection burden, and also to present a snapshot of their prevalence patterns in a large sample of Croatian children with RTIs, evaluating viral seasonality as well.

## An Overview and Global Trends

### Seasonal Coronaviruses

Epidemiological understanding of seasonal coronaviruses (sCoVs) is currently incomplete in many settings around the world, primarily due to the fact that these viruses are not a part of standard diagnostic armamentarium, or testing is guided by specific clinical case definitions (6). It is well-established that sCoVs are endemically found in co-circulation with other prevalent respiratory viruses, which is a principal reason why co-infections are commonly observed (7–9). Furthermore, the habitual occurrence of sCoVs during periods characterized by high influenza activity underscores the significance of their consideration within the context of viral respiratory infections (9–11). Likewise, during the COVID-19 pandemic, co-circulating viruses may be responsible for symptoms that resemble the clinical presentation of COVID-19, which then poses a problem on how to quickly establish a correct diagnosis in settings without the capacity for multiplex testing (9).

Four sCoV strains can give rise to cold symptoms in human individuals and are responsible for 15–30% of respiratory infections every year: 229E-CoV, NL63-CoV, OC43-CoV, and HKU1-CoV (12, 13). Regardless of the fact that they utilized different host receptors for cell entry, all sCoVs express the spike glycoprotein (which protrudes from the surface of the virions), with high homology pattern between 229E-CoV and NL63-CoV (14). This spike glycoprotein is comprised of two subunits: S1 harbours the receptor-binding domain responsible for binding to cell surface receptors, while S2 is pivotal for mediating the fusion of viral and host membranes and subsequent cell entry (15). It has to be noted that, in comparison to S1 that is more variable, certain portions of the S2 subunit represent the most conserved part of the molecular structure among both sCoVs and zoonotic coronaviruses (including SARS-CoV-2) (12, 16).

The increased availability of reverse-transcriptase polymerase chain reaction (RT-PCR) enabled much easier detection of the infection for each of the four aforementioned sCoVs (17). Nonetheless, as we gained more valuable data from population-based studies that utilized molecular methods, there was still not much attention paid to these viruses and their possible role in various clinical presentations, possible because very little was known regarding their exact role in respiratory illnesses (15, 18, 19). And while recent studies on hospitalized patients have confirmed the important role of sCoVs and their global circulation (20), we still lack pertinent data on the frequency and seasonality in the broader community context (21).

However, a recent systematic review by Park et al. (22) revealed a consistent winter peak of sCoV incidence in the northern hemisphere (akin to other respiratory viruses), with moderate decrease during the summer months. It has to be noted that there is a substantial variation in the percentage of sCoVs infections in different epidemiological studies that appraised patients with acute respiratory infections. Heimdal et al. (23) followed Norwegian children hospitalized with respiratory infection for 9 years, and revealed that sCoVs were implicated in 9.1% of the episodes. Among different coronaviruses, OC43-CoV was the most commonly found, while 229E-CoV was the rarest, with most of the infections occurring during winter months (23).

For some time now we know that, in contrast to the observed situation with COVID-19, infection rates of sCoVs are much higher in children and adolescents. This is especially evident in a recent cohort study from Michigan in United States, that demonstrated the highest infection rates of sCoVs in children younger than 5 years of age (18 per 100 person-years) when compared to older age groups (7–11 per 100 person-years) (18). Children have little or no pre-existing immunity against sCoVs, and they are often in close contact in nurseries and childcare settings (12). Other interesting finding is that seasonal sCoVs infections in younger individuals may actually act as a protecting factor from symptomatic/severe SARS-CoV-2 infections by limiting viral infection, since large amounts of cross-reactive antibodies between sCoVs and SARS-CoV-2 have been found in this age group (16).

Some other studies point toward the important role of sCoVs in younger age groups. A cross-sectional, prospective study on a convenience sample of 1,404 Mexican children with community-acquired pneumonia demonstrated the presence of sCoVs in 2.2% of all the samples (24). Conversely, the prevalence was much larger in paediatric patients with cancer in a three-year retrospective study from Turkey, where sCoVs were the third most prevalent viral group (after rhinovirses and parainfluenza viruses) with the prevalence of 14.8% (25). In a recent big study from Scotland, the prevalence of sCoVs was 4.0% among all the tested patients (i.e., 74 519 of them), contributing to 10.7% of all respiratory virus detections (11). In the latter study the most prevalent detection was OC43-CoV, while the prevalence of HKU1-CoV was very low (i.e., 0.3% overall).

In a study from Russia on 1560 children with upper or lower respiratory infection, sCoVs were found in 0.8% of positive samples (26). On the other hand, a large retrospective observational study from Moscow found sCoVs in 2.6–6.1% of individuals with acute respiratory infection (ARI) between January 2016 and March 2020 (i.e., prior to SARS-CoV-2 pandemic), with winter-spring seasonal activity pattern and peak levels in December (27).

Interestingly, a recent study has found that sCoVs activity in the temperate regions of China seems to be less seasonal, with high activity observed in the summer, autumn and winter (28). Moreover, a large retrospective study from Beijing showed that sCoVs are present in 1% of clinical samples from adults with ARI (29), while a prominent 12-month prospective study from Hong Kong demonstrated that a total prevalence of sCoVs in patients with ARI was 2.1% (with HCoV-NL63 showing a highest positivity rate of 1.3%) (30).

Many of these studies highlight the potential of interactions and co-infections between sCoVs and other respiratory viruses. An in-depth analysis by Nickbakhsh et al. (11) validates positive interactions at the sCoV type level. On the other hand, the evidence of immunological cross-protection between different human coronaviruses is lacking, with inconsistent reports of antigenic cross-reactivity demonstrated by some studies (31, 32), but not confirmed by other ones (33). Genetic relatedness of coronaviruses enables the cross-reactivity at the genus level (34), but more general cross-reactivity between 229E-CoV and OC43-CoV has also been described (35).

In any case, serological surveys on the population level will be indispensable for establishing true sCoV infection burden and the respective age distribution, as well as appraising the prospect of coveted cross-protective immunity. Findings from a recent study by Fischer et al. endorse the use of national influenza surveillance systems for sCoV early detection and monitoring, which will enable a an enhanced estimation of the burden of disease, and also aid in tracking emerging coronaviruses such as SARS-CoV-2 (20).

### Rhinoviruses

Rhinoviruses are the most common cause of ARI in all age groups (36–39), which poses a significant burden for the health care system, as well as a substantial economic loss caused by absenteeism (40). Although they lead in the aetiology of ARI, they have been neglected for years for a number of reasons: (i) they were seen as rare (or improbable) causative agents of lower respiratory tract infections (LRTIs) since their replication is difficult at temperatures above 33°C; (ii) because they cause self-limiting upper respiratory tract infections (URTIs) in healthy individuals, do not cause hospitalization and, consequently, do not burden the hospital system; (iii) until the development of molecular detection methods the laboratory diagnosis of these viruses was relatively slow, therefore clinically irrelevant and expensive, and; (iv) to date there is no commercially available effective specific antiviral therapy or vaccine specifically targeting rhinovirus (41). The increased availability of molecular detection methods, together with the fact that rhinoviruses can replicate efficiently at lower airway temperatures (42), allowed rhinovirus infections to be viewed from a different vantage point.

Rhinoviruses are estimated to cause more than half of URTIs usually presenting as common cold syndrome; however, during the last two decades, numerous studies have revealed their role as leading causes of LRTIs (38, 39, 43, 44). A recent prospective study conducted in 11 European countries has shown that in adults presenting to primary care with LRTI, the most common viral pathogens detected were human rhinoviruses (20.1%) (45). Another recent study conducted on hospitalized children with ARI in Croatia also revealed rhinovirus as the most frequently detected virus, diagnosed in 33.4% patients; 60.4% as monoinfection, and 39.6 % as co-infection with other respiratory viruses (46). More than half of children infected with rhinovirus (55.8%) presented with LRTI (46).

The high prevalence of rhinoviruses each year is not surprising, given the enormous diversity of these viruses as a result of a large number of serotypes/genotypes. After overcoming the infection, serotype-specific humoral immunity important for preventing the infection is induced, with little or insignificant cross-neutralization among serotypes (47, 48); hence, infection with different serotype can frequently occur. Rhinoviruses antigenic diversity is also major obstacle in vaccine development (49).

Currently, ~170 genotypes (50) are classified into three species: Rhinovirus A (RV-A), Rhinovirus B (RV-B), and Rhinovirus C (RV-C) under the Enterovirus genus. Current classification is based on capsid region sequences analysis (VP4/VP2 or VP1), since sequencing the 5′ untranslated region (5′UTR) cannot discriminate between all rhinovirus species (particularly RV-A and RV-C) (30).

In addition to relevant differences in genetic sequence, there are several phenotypic key differences between species. Depending on the species, rhinoviruses attach to the different host cell receptor: most HRV-A and HRV-B attach to the intercellular adhesion molecule (ICAM)-1, the others alternatively bind to low density lipoprotein receptor (LDL-R), whereas RV-C utilizes human cadherin-related family member 3 (CDHR3) (51). Furthermore, RV-A and RV-B can be cultured in cell cultures while RV-C cannot be grown, which is why the latter species was discovered much later than RV-A and RV-B (52).

The currently unresolved issue that is the focus of scientific interest is whether a particular species of rhinovirus causes a more severe illness compared to another species. In any case, the data is controversial. Some studies have recorded an association of severe disease with HRV-C (53), and to a lesser extent with HRV-A (54), while other research groups have not corroborated this association (55). The complexity of these observations is emphasized by the finding that the relationship between species and disease severity also depends in some extent on age, i.e., it is frequently noted that RV-C tends to cause more severe disease in children (56) and HRV-A in adults (54). Most studies are however in agreement that RV-B tends to occur sporadically and often in asymptomatic patients (46, 54, 57).

In an already mentioned study from Russia that included children with URTIs or LRTIs, rhinovirus was found in 15.1% of positive samples (26). Likewise, a study in adults demonstrated the presence of rhinovirus in 15.5% of patients with positive microbiology results (58). There was a high prevalence of rhinovirus infection in paediatric patients with ARI (59), while a large study from the CAP-China Network revealed its presence in 1.8% of adult patients with community-acquired pneumonia—although its potential role in the pathogenesis is still not clear (60). In addition, a recent meta-analysis did not find any significant difference in the prevalence between children of different age groups, or those with severe disease in comparison to asymptomatic ones (61). Consequently, until the true role of rhinovirus in more severe disease presentations is established, it is hard to make any steadfast recommendations regarding surveillance and clinical approach.

Since the discovery of rhinovirus, many longitudinal studies have shown that rhinoviruses can be detected throughout the year (62), with the awareness that in countries with temperate climate they occur more frequently in autumn and spring (63). Recent studies from Croatia have also shown this pattern of rhinovirus prevalence with peak of rhinovirus prevalence detected during autumn and spring, while influenza viruses, respiratory syncytial virus, and metapneumovirus predominate in the winter (46, 64). However, some studies indicate that severe rhinovirus infection are more common in the winter (65), possibly due to a weaker induction of interferon at low temperatures, resulting in less efficient antiviral defence response of infected cells (66). There are also differences in the occurrence of individual species, i.e., some studies have observed that HRV-C usually occurs in winter months (46, 67).

### Adenoviruses

Human adenovirus (HAdV) is associated with a wide range of illnesses, ranging from common cold to more serious conditions—including serious acute respiratory infections, gastroenteritis, conjunctivitis, haemorrhagic cystitis or meningoencephalitis, which are often underreported (68). Its paramount role as an agent of RTIs is especially pertinent for children between 1 and 5 years of age (69, 70), primarily due to their immature immune system (71). The vast majority of cases are asymptomatic and self-limited (72); nonetheless, the clinical spectrum is broad, and dissemination or pneumonia can be fatal, both in immunocompetent and immunocompromised patients (73, 74).

The virus is spread via aerosolized droplets, direct inoculation to the conjunctiva, exposure to infected tissue/blood, as well as via faecal-oral route (72). There is also a possibility of viral acquisition from exogenous sources (e.g., pillows, linens, lockers, guns) or viral reactivation (75). It is important to note that AdV RTIs normally occur all-year-round (62), unless there is an epidemic outbreak (70, 76). In such cases connections between certain periods of the year and the incidence of AdV RTIs can be drawn.

For example, one study finds that in Brazil the adenovirus is more active during the rainy season with higher air humidity (77). Another study demonstrated that HAdV circulated year-round, with higher frequency during winter and early spring; increases in the average monthly temperature were associated with decreases in HAdV infections (70). Other studies have found HAdVs throughout the year, with a notably higher prevalence in summer (78).

In a majority of studies predominant clinical symptoms in the HAdV-infected children were fever, cough and pneumonia (68)—however, clinical presentation alone is usually not sufficient for establishing a correct diagnosis. As most cases of acute respiratory infection show similar symptoms regardless of the causative viral agent, correct diagnosis usually relies on laboratory confirmation (78). When multiplex PCR is used as a diagnostic tool in RTI, the rate of adenoviral infections is between 20 and 36% (69, 79, 80). Positive adenovirus PCR accompanied with a higher viral load is associated with more severe symptoms and worse prognosis, which is when the early use of cidofovir may improve the outcome (78).

Moreover, viral–bacterial co-infections frequently occur, and a number of research studies underscore the potential risk of synergistic presentation during the co-infection process with respiratory viruses and bacteria, resulting in longer hospital stays and higher morbidity (81). These co-infection patterns dominate among children when compared to the adults; more specifically, they account for 35% of cases among paediatric patients, and only 5.8% among adult ones (80). Also, it has to be taken into account that a substantial mortality burden can be attributed to secondary bacterial infections from Streptococcus pneumoniae or Staphylococcus aureus (81).

Diverse clinical presentations of adenoviral infections can be epidemiologically linked to various genotypes (81). These genotypes show diverse tissue tropisms that are linked with the manifestation of infection. For example, HAdV species B (which includes HAdV-3, 7, 11, 14, 16, 21, 50 and 55), species C (which includes HAdV-1, 2, 5 and 6) and species E (which includes HAdV-4) are predominantly related to respiratory diseases (82, 83) and, thus, of interest for researchers and clinicians alike. A large retrospective cohort study from Monroe Carell Jr. Children's Hospital at Vanderbilt revealed that HAdV species C and HAdV species B were the most frequent ones, with notable differences in clinical manifestations and outcomes (84).

Other recent studies have shown that the three predominant genotypes in children younger than 5 years of age were HAdV-3, HAdV-7 and HAdV-2 (85). The patients infected with the HAdV-2 genotype were also accompanied with leucocytosis. Furthermore, two genotypes have a much higher chance of infecting young children as well as causing more severe symptoms, and those are HAdV-1 and HAdV-2 (86). A study from Switzerland singled out HAdV8 as a predominant genotype causing HAdV infection among young adults, middle-aged and elderly, and HAdV1-3 as predominant genotypes causing HAdV infection among young children (68). In a study from Kuwait, HAdV C1, C2, C5, B3, and B7 were recognized as the main types identified in patients with severe respiratory infection (87).

Furthermore, in a large study from Russia that analysed respiratory tract samples of 4,731 patients, HAdV infection has been detected in 6.9% of diseased children and 2.9% of adults year-long, with a notable peak in October-December (88). Another study demonstrated the presence of HAdV in 3.5% of positive samples in Russian children with RTI (26). In children that were hospitalized due to severe ARI in Beijing and Shanghai, the prevalence of HAdV was 13.7% (89). A very recent study from Macao on hospitalized children with ARI showed even higher prevalence of HAdV of 15.8%, with infection peaks in summer and winter (90).

Nearly all adenovirus infections with high morbidity and severe symptoms in children are associated with HAdV-7 instead of HAdV-3 (91). One notable example is China, where HAdV-7 is one of the predominant genotypes, accounting for 26.9% of all adenoviral infections (and also responsible for a myriad of outbreaks) (83, 92). Still, all these type-specific studies of adenoviral infections come with certain limitations, as we are not dealing with only a mechanistic process of infection, but also a panoply of host and environmental factors.

### Bocaviruses

Since it has been initially discovered 16 years ago in nasopharyngeal samples of children with ARI, it quickly became evident that human bocavirus (HBoV)—and more specifically HBoV type 1 (HBoV 1)—can be considered an important respiratory pathogen (93, 94). Not much later, three other bocaviruses have been discovered and consequently named HBoV 2-4; nevertheless, their role in clinical disease remains somewhat controversial. Even from the more fundamental perspective, some investigators hypothesize that HBoV infection under clinical conditions may depend on helper viruses, such as herpesviruses, or that HBoV replicates utilizing a mechanism atypical for parvoviruses (95). These insights could at least partially contribute to understanding the high burden of co-detection of HBoV with other viruses.

HBoV is classified into Parvoviridae family and Bocaparvovirus genus, with two species that infect humans: Primate bocaparvovirus 1 that contains HBoV 1 and HBoV 3, and Primate bocaparvovirus 2 containing HBoV 2 and HBoV 4 (93, 96). Akin to other parvoviruses, HBoV is most likely transmitted via droplets and aerosol, while the total global prevalence of HBoV was estimated at 6.3% (93). Seroepidemiological studies revealed that, by the age of six, 90–100% of children have circulating antibodies against at least one of the four human bocaviruses (97). Later, HBoV1 IgG antibody concentrations remain high during adulthood, probably because of the “immunity boost” caused by circulating HBoV1, or by an infection with related HBoV2, HBoV3, or both (93, 97). Primary bocavirus infections mostly arise between 6 and 24 months of age, which is later than RSV infections, but earlier in childhood than influenza (97).

When country-specific data is considered, a Croatian study revealed a high rate of HBoV1 among infants and small children with lower respiratory tract infection that required hospitalization (i.e., 23.1% of those with proven viral aetiology) (98), while the other study showed that two thirds of HBoV positive patients were between 1 and 3 years of age (99). In Egypt, the prevalence of HBoV in nasopharyngeal swabs taken from children with acute respiratory tract infection was 9.3% (100). Furthermore, HBoV was detected in 1.9% of the patients in Kuwait, with a peak incidence among children <1 year of age, as well as the predominance of HBoV-1 genotype (101). In a Belgian study, HBoV was detected in 5.7% of children with a median age of 10.6 months (102).

A recent study from Russia found HBoV in 5.8% of positive samples in children with RTI (26). On the other hand, the prevalence can be substantially higher in children hospitalized with severe ARI, as demonstrated by a recent study on hospitalized children in Beijing and Shanghai (i.e., encompassing both northern and southern China), where the prevalence was 19.1% (89).

In any case, it is evident that the role of HBoV 1 as a respiratory pathogen is rather well-established, so nowadays the virus is generally acknowledged as an important player in both upper and lower acute respiratory disease. In children, the virus is linked to rhinitis, acute otitis media, pneumonia, bronchiolitis, and asthma exacerbations (97). One large meta-analysis showed that HBoV 1 is the third most common viral agent detected in children with bronchiolitis (103), while other investigators placed HBoV as the third most common virus in children suffering from wheezing with prevalence of 8.1%, following rhinoviruses and respiratory syncytial virus (104). Due to its non-enveloped nature, HBoV is resistant to disinfectants and detergents, and should be considered as a possible nosocomial pathogen (which is especially pertinent for immunocompromised children) (105).

A few case reports have been published describing extrapulmonary manifestations in children infected with HBoV 1-3, such as encephalitis, hepatitis and myocarditis (97, 106–108); however, clinical presentation in other age groups remains an open question. One study showed that HBoV 1 can also be found in immunocompetent adults with respiratory infection, where it can be associated with a high incidence of pneumonia—especially in elderly individuals and patients with nosocomial infections (109). On the other hand, a study among adult patients with severe pneumonia necessitating ICU admission demonstrated that monoinfection with HBoV 1 is not common finding, i.e., there is a significant burden of co-infections with other well-established respiratory pathogen (110).

This co-infection pattern with other respiratory pathogens may arise due to the prolonged shedding and possible persistence, especially in children. It is well-established that asymptomatic children can shed virus more than 1 month and in prolonged duration up to 1 year (111). However, even in respiratory samples containing actively transcribing HBoV1, other viruses have been detected in almost 60% of the cases (97). Moreover, a recent study from Italy showed co-infection of HBoV in 51.7% of patients (112). The true significance of such co-detection or co-infection is yet to be determined, which hampers our attempts to address true pathogenic potential of bocavirus (113).

The diagnostic approach is another hurdle. Due to the difficulties in replicating the virus in cell cultures, and the fact that the serology of bocavirus is complicated by the phenomenon known as the “original antigenic sin,” the diagnosis of HBoV infection is almost exclusively based on molecular detection methods (93, 97, 100). Most laboratories currently use in-house PCR and real-time PCR assays targeting the NP-1, NS-1 or VP1/2 gene, but other nucleic acid-based detection methods for the diagnosis of HBoV have also been described (114). However, clinical value of PCR detection is low due to prolonged shedding and common co-detection with other viruses. Therefore, the other diagnostic strategies are needed. Albeit a point-of-care test detecting viral antigen has been used (115), additional evaluation of this test was not pursued.

## Local Prevalence Patterns—A Snapshot of the Viral Seasonality in Croatia

The aforedescribed global overview is indispensable for placing any local data into the appropriate context, especially taking into account global prevalence ranges informed by the detailed literature review (Table 1). As the ongoing COVID-19 pandemic hampered many regular epidemiological endeavours, data gathered just prior to its emergence will be increasingly used to inform our further epidemiological approaches in this field. Consequently, a recent study conducted between May 2017 to April 2019, on 590 individuals younger than 18 years of age (median age 1.75 years; range 7–17), may be used as an informative blueprint. This epidemiological study utilized a multiplex RT-PCR for the detection of 15 respiratory viruses in nasopharyngeal and pharyngeal flocked swabs (46).

**Table 1:** Taxonomy and global prevalence of four viral groups (seasonal coronaviruses, rhinoviruses, adenoviruses and bocaviruses) in individuals with respiratory tract infections (RTIs).

**Virus**	**Family**	**Genome**	**Subtypes**	**Global prevalence in RTIs %**
Seasonal coronavirus (sCoV)	*Coronaviridae*	ss(+)RNA	229E-CoV, NL63-CoV, OC43-CoV, HKU1-CoV	0.5–18.4
Rhinovirus	*Picornaviridae*	ss(+)RNA	3 species (A–C), >100 serotypes	13–59
Human adenovirus (HAdV)	*Adenoviridae*	dsDNA	7 species (A–G), >80 genotypes	1–36
Human bocavirus (HBoV)	*Parvoviridae*	linear ssDNA	HBoV 1–4	1–56.8

*ss, single stranded; ds, double stranded; (+), positive sense; CoV, coronavirus; sCoV, seasonal coronavirus; HAdV, human adenovirus; HBoV, human bocavirus*.

The male to female ratio in the study was 1.42:1; furthermore, the upper respiratory tract infection has been established in 46.9% patients, while lower respiratory tract infection was found in 53.1% of patients. Pursuing a comprehensive panel of respiratory viruses on this population had both epidemiological and clinical merit, as the results of virology testing were sent to the attending physicians once per week. In a total of 76.4% of patients there was a proven viral infection; 69.8% of positive patients had a monoinfection with a single virus, while 30.2% of them harboured two or more viruses synchronously (46).

In any case, this epidemiological analysis (covering north-western and central part of Croatia) revealed that rhinoviruses were the most commonly detected group with a prevalence of 33.4%. Adenoviruses were the third most common group (after respiratory syncytial virus, prevalence of 15.6%), seasonal coronaviruses were on the sixth place (following influenza viruses and parainfluenza viruses, prevalence of 7.1%), and bocaviruses were placed immediately after seasonal coronaviruses (prevalence of 5.3%) (46). Hence, albeit overlooked, the clinical importance of these viruses is becoming increasingly evident.

**Figure 1:**
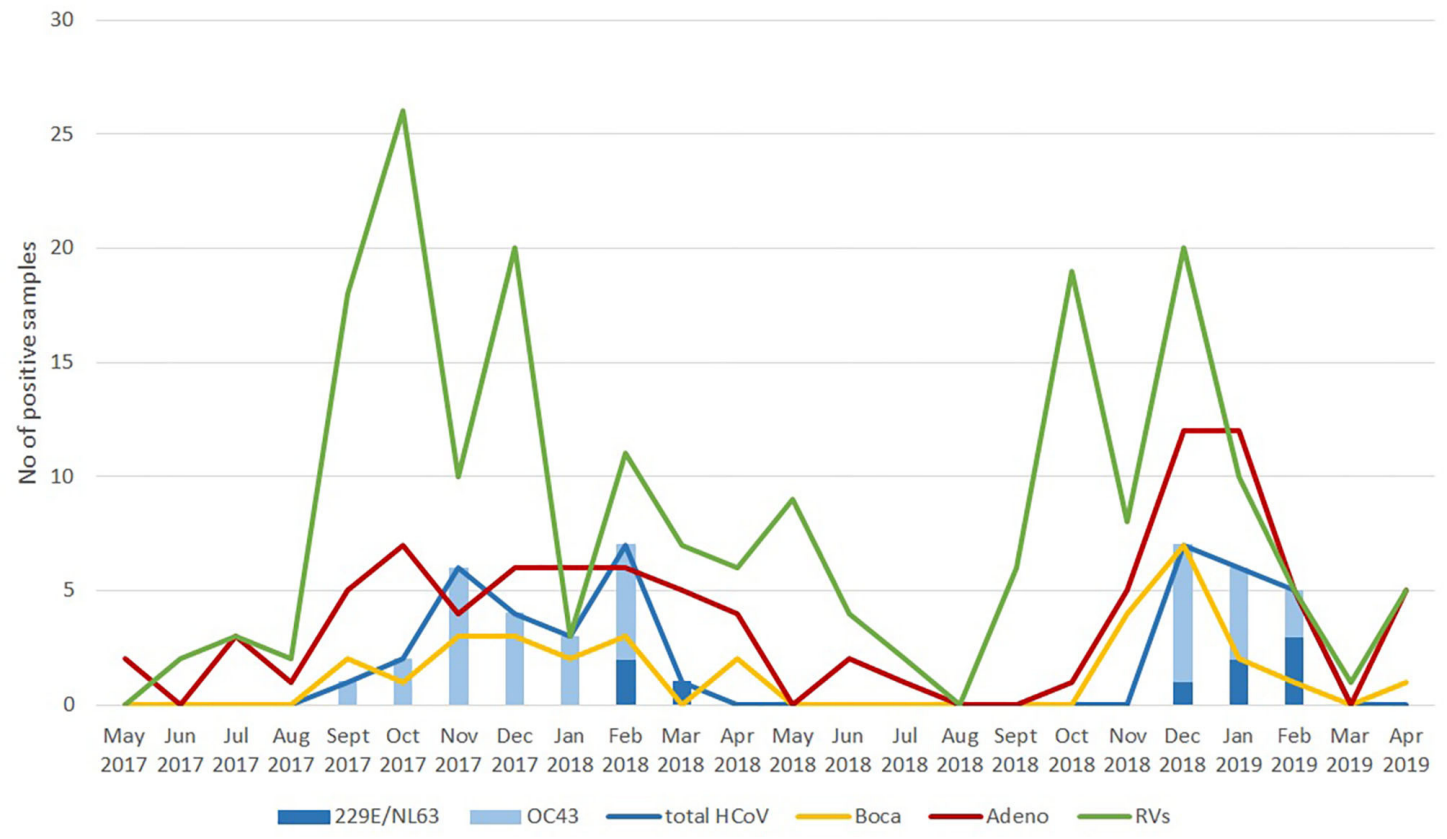
Temporal distribution of cases positive for seasonal coronaviruses, rhinoviruses, adenoviruses, and bocaviruses in hospitalized Croatian children with acute respiratory infection from May 2017 to April 2019.

As a follow-up to this review, we also argue that there is a need to always take into account the seasonality of neglected (or unappreciated) respiratory infections. This is the reason why we decided to present here novel graphical data on temporal distribution of seasonal coronavirus, rhinovirus, adenovirus and bocavirus positive cases in these 590 hospitalized children within a 2-year period (Figure 1). Although the prevalence is expectedly the highest in the winter period, it is clearly visible that seasonality of different viral groups may differ. Also, from the graphical data it is clear that the most common seasonal/endemic coronavirus is HCoV-OC43 (Figure 1).

## Conclusions

Timely detection of seasonality of some respiratory viruses (enveloped viruses such as seasonal coronaviruses) in the local context can help the targeted and cost-effective use of viral diagnostics. For the other, non-enveloped, year-round viruses (i.e., rhinovirus, adenovirus and bocavirus), continuous virological diagnosis needs to be implemented in clinical laboratories to more effectively address the aetiology of respiratory infections and assess the impact of these viruses on disease burden. As these viral groups still represent a significant global infectious burden, they should not be neglected—even when all research and clinical efforts seem to be devoted to COVID-19. In conclusion, appraising the exact prevalence of (often neglected) respiratory viruses is indispensable for adequate prevention, control and therapeutic approaches to RTIs.

## Author Contributions

SL-S and TM conceptualized and designed the manuscript. SL-S, TM, IL, and MM conducted epidemiological investigation and data curation. SL-S, TM, IL, and MM interpreted the data and prepared the draft of the manuscript. SL-S and TM created visualizations. JV supervised the project, and JV and SL-S critically reviewed the draft. Project administration was done by SL-S. All authors have read and agreed to the published version of the manuscript.

## Conflict of Interest

The authors declare that the research was conducted in the absence of any commercial or financial relationships that could be construed as a potential conflict of interest.
